# Novel functions of LHX2 and PAX6 in the developing telencephalon revealed upon combined loss of both genes

**DOI:** 10.1186/s13064-017-0097-y

**Published:** 2017-11-15

**Authors:** Geeta Godbole, Achira Roy, Ashwin S. Shetty, Shubha Tole

**Affiliations:** Department of Biological Sciences, Tata Institute of Fundamental Research, Mumbai, India

**Keywords:** Organizer, Dorsoventral, Patterning, Craniofacial, Mediolateral, Hem

## Abstract

Patterning of the telencephalic neuroepithelium is a tightly regulated process controlled by transcription factors and signalling molecules. The cortical primordium is flanked by two signalling centres, the hem medially, and the antihem laterally. The hem induces the formation of the hippocampus in adjacent neuroepithelium. Therefore, the position of the hem defines the position of the hippocampus in the brain. The antihem is positioned at the boundary between the dorsal and ventral telencephalon and proposed to provide patterning cues during development.

LIM-homeodomain (LIM-HD) transcription factor LHX2 suppresses both hem and antihem fate in the cortical neuroepithelium. Upon loss of *Lhx2*, medial cortical neuroepithelium is transformed into hem, whereas lateral cortical neuroepithelium is transformed into antihem. Here, we show that transcription factor PAX6, known to regulate patterning of the lateral telencephalon, restricts this tissue from transforming into hem upon loss of *Lhx2*. When *Lhx2* and *Pax6* are both deleted, the cortical hem expands to occupy almost the complete extent of the cortical primordium, indicating that both factors act to suppress hem fate in the lateral telencephalon. Furthermore, the shift in the pallial-subpallial boundary and absence of the antihem, observed in the *Pax6* mutant, are both restored in the *Lhx2; Pax6* double mutant.

Together, these results not only reveal a novel function for LHX2 in regulating dorsoventral patterning in the telencephalon, but also identify PAX6 as a fundamental regulator of where the hem can form, and therefore implicate this molecule as a determinant of hippocampal positioning.

**Electronic supplementary material:**

The online version of this article (10.1186/s13064-017-0097-y) contains supplementary material, which is available to authorized users.

## Introduction

In development, tissues are patterned by the action of morphogens secreted by signaling centers known as “organizers.” The WNT- and BMP- secreting cortical hem in the telencephalon is one such medial signaling center [1, 2]. At the lateral edge of the cortical neuroepithelium, the EGF-expressing antihem defines the boundary between the dorsal and ventral telencephalon [3].

Previously, we demonstrated that the hem is capable of organizer function, by creating embryos with multiple hems in the telencephalon, and showing that each ectopic hem induces the formation of an ectopic hippocampus [4]. The factors that act to restrict the hem to its position at the medial edge of the cortical neuroepithelium are therefore critical determinants of where the hippocampus will form in the brain. Transcription factor LHX2 is a critical suppressor of the cortical hem. In the *Lhx2* null mutant, the cortical primordium cannot retain its identity, but is transformed into two alternate fates: medial cortical neuroepithelium is transformed into hem, whereas lateral cortical neuroepithelium is transformed into antihem [4, 5]. In mosaic experiments, *Lhx2* null clones in the medial telencephalon display hem fate, and those in the lateral telencephalon display antihem fate, indicating a cell-autonomous requirement for LHX2 in cortical neuroepithelial cells [4]. Why loss of *Lhx2* leads to two disparate fate transformations in the dorsal telencephalon is unclear.

Paired domain containing homeobox transcription factor PAX6 is required for antihem fate, which is lost in *Pax6* null mutants [3]. *Pax6* is expressed in a lateral(high)-medial(low) gradient, opposite to that of *Lhx2* [6, 7]. We hypothesized that the presence of PAX6 may restrict the medio-lateral extent of the hem in *Lhx2* mutants. Supporting this hypothesis, in the mosaic experiments performed in Mangale et al. (2008), PAX6 was lost in medial *Lhx2* null patches that formed ectopic hems, but not in lateral *Lhx2* null patches that formed antihem. Here, we examined medio-lateral patterning in embryos lacking both *Lhx2* and *Pax6*, using null mutants as well as conditional knockout strategies. We find that the hem indeed expands to encompass almost the entire dorsal telencephalon when both *Lhx2* and *Pax6* are lost. Concomitantly, the loss of both factors appears to partially restore dorsoventral patterning that is disrupted in the *Pax6* single mutant [8]. Thus, an examination of the *Lhx2;Pax6* double mutant uncovers novel functions for both transcription factors, not seen upon loss of either factor alone. Our results show LHX2 to be a regulator of dorsoventral patterning in the telencephalon. PAX6 emerges as a key player in controlling the medial position of the hippocampus, because it prevents the hippocampal organizer, the hem, from being formed at lateral positions.

## Results and discussion

We used an *Lhx2* null mutant line generated by targeted gene disruption [9] and the *Pax6* ^*sey/sey*^ line which has a point mutation in the *Pax6* gene creating a null allele [10] to generate the *Lhx2* ^*−/−*^ *;Pax6* ^*sey/sey*^ double null mouse mutant. This mutant suffers from gross craniofacial abnormalities including an absence of external eyes, similar to the individual null phenotypes for *Lhx2* ^*−/−*^ *and Pax6* ^*sey/sey*^ embryos, and also lacks external nostrils similar to *Pax6* ^*sey/sey*^ [9, 11]. A unique feature in the double null is the presence of cleft-lip/palate, indicating a combinatorial role for LHX2 and PAX6 in craniofacial development (Additional file 1: Figure S1). To our knowledge, this is the first characterization of an *Lhx2;Pax6* double null mutant.

## PAX6 and LHX2 together restrict medial cortical fate to the medial telencephalon

**Fig. 1:**
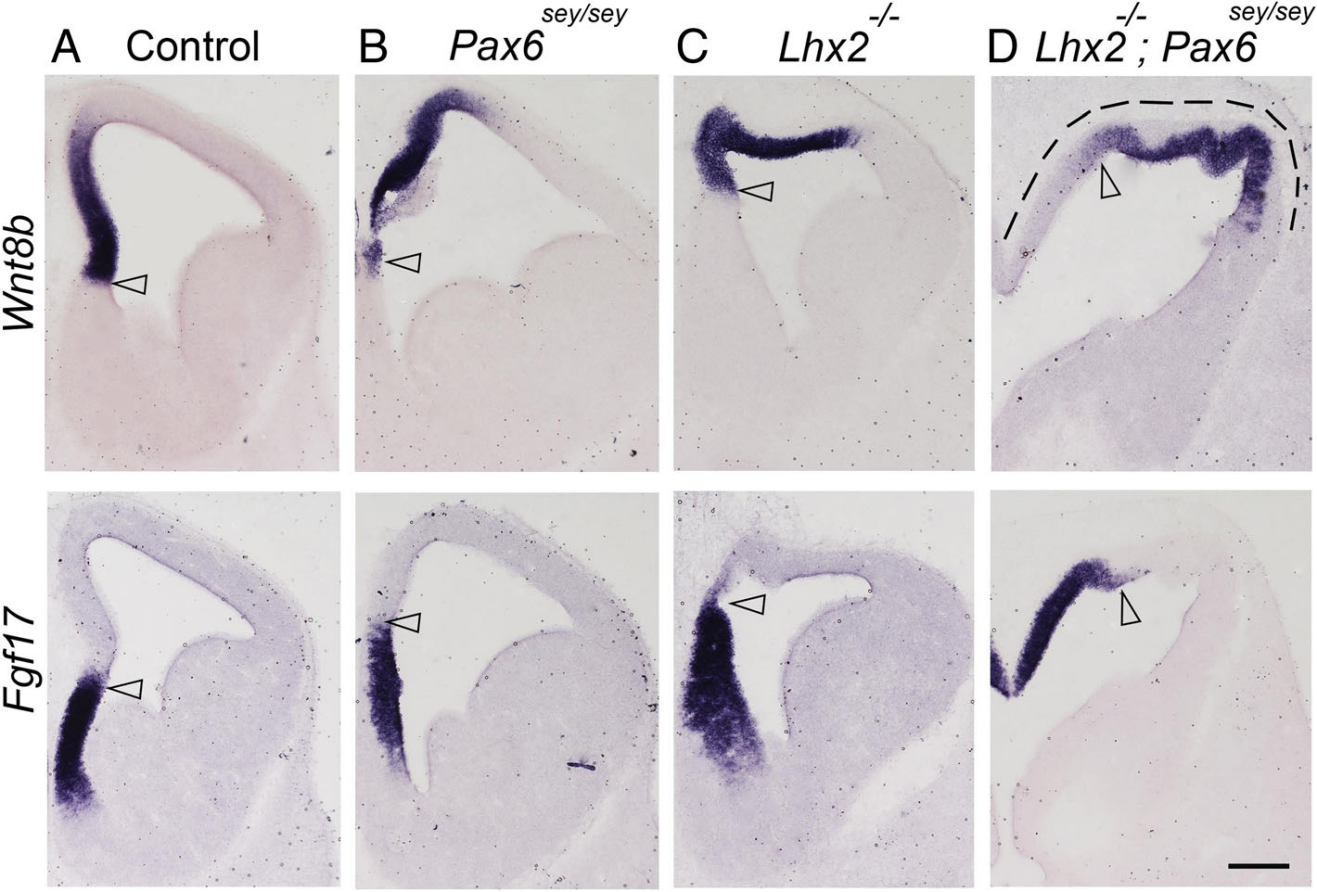
Medial fates are expanded laterally upon loss of both LHX2 and PAX6. **a**-**d** E12.5 coronal sections of Control (**a**), *Pax6* ^*sey/sey*^ (**b**), *Lhx2* ^*−/−*^ (**c**), *and Lhx2* ^*−/−*^ *;Pax6* ^*sey/sey*^ (**d**) were examined for the expression of medial cortical marker *Wnt8b* and septum marker Fgf17, which identify adjacent, non-overlapping regions of the medial telencephalon (open arrowheads mark the boundary between the two markers). In the double mutant, these medial markers together encompass the entire dorsal telencephalon (dashed line, **d**). Scale bar is 200 μm

We analyzed *Lhx2* ^*−/−*^ *;Pax6* ^*sey/sey*^ double mutants at embryonic day (E)12.5 with markers of medial telencephalic structures and compared the results with the expression seen in the respective single null mutants. We examined medial cortical marker *Wnt8b* which identifies the hem and the hippocampal primordium, and septum marker *Fgf17*. As expected, *Wnt8b* expression marks the medial telencephalic primordium in the *Pax6* ^*sey/sey*^ mutant. In the *Lhx2* ^*−/−*^ mutant, *Wnt8b* is expressed in the expanded hem, sparing some lateral tissue. In contrast, loss of both genes results in the entire dorsal telencephalon expressing medial markers, apparently sparing no lateral cortical neuroepithelium (Fig. 1). However, the double null mutant brains were fragile and the morphology made it difficult to analyze this apparent lateral-to-medial transition properly. Furthermore, the individual null mutants of *Lhx2* and *Pax6* are embryonic lethal [9, 10], and both genes lie on chromosome 2, which makes it difficult to obtain homozygous *Lhx2* ^*−/−*^ *;Pax6* ^*sey/sey*^ double null mutants from heterozygous crosses. Therefore, we generated *Lhx2* ^*lox/lox*^ *;Pax6* ^*lox/lox*^ double conditional mutants and crossed them with a ubiquituously expressed tamoxifen inducible *CreER* line.

## *Lhx2* and *Pax6* double conditional mutants phenocopy the patterning defects in the corresponding double null mutants

**Fig. 2:**
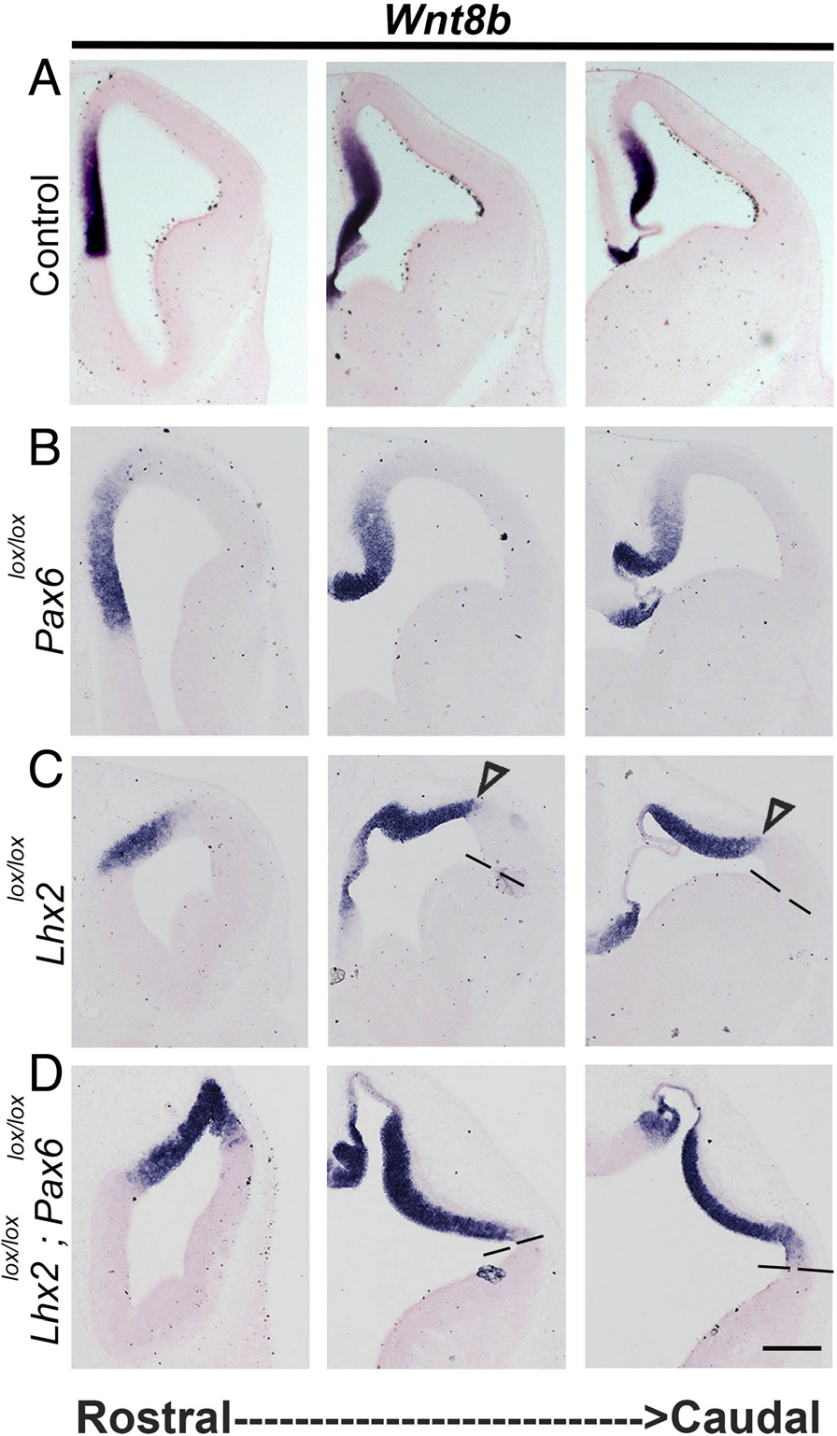
Conditional deletion of *Lhx2* and *Pax6* from E8.5 recapitulates the lateralization of medial fates seen in the double null mutants. **a**-**d** Tamoxifen was administered at E8.5 to Control (**a**), *CreER;Pax6* ^*lox/lox*^(**b**), *CreER;Lhx2* ^*lox/lox*^(**c**), *and CreER;Lhx2* ^*lox/lox*^ *;Pax6* ^*lox/lox*^ (**d**) animals, and the embryos were harvested at E12.5. Coronal sections were examined for *Wnt8b* expression at rostral, mid, and caudal levels of sectioning. *Wnt8b* expression is restricted to the medial cortical neuroepithelium in control and *Pax6* ^*lox/lox*^ brains (**a**, **b**). In *Lhx2* ^*lox/lox*^ brains, a small region of lateral neuroepithelium remains *Wnt8b*-negative (region between open arrowhead and dashed line, **c**). In the double conditional mutants, *Wnt8b* expression extends almost upto the pallial-subpallial boundary (dashed lines, **c**, **d**). Scale bar is 200 μm

We tested whether administration of tamoxifen at E8.5 to *CreER*;*Lhx2* ^*lox/lox*^ *;Pax6* ^*lox/lox*^ animals recapitulates the results we obtained from the respective single and double null mutants by E12.5. For all experiments using *Lhx2* and *Pax6* single or double conditional mutant embryos, we examined the expression of the respective floxed exon(s) in each embryo in serial sections, and only used embryos which displayed extensive recombination for analysis. One example of an *Lhx2* ^*lox/lox*^ *;Pax6* ^*lox/lox*^ double conditional mutant brain is shown in Additional file 2: Figure S2. Loss of *Pax6* is not reported to affect patterning of the dorsomedial telencephalon [12] and indeed, the extent of *Wnt8b* expressing neuroepithelium appears comparable in *Pax6* ^*lox/lox*^ and control embryos (Fig. 2a, b). In *Lhx2* ^*lox/lox*^ embryos, *Wnt8b* expression appears to spare some lateral telencephalic tissue, which is presumably the antihem [4]. In the *Lhx2* ^*lox/lox*^ *;Pax6* ^*lox/lox*^ double conditional mutant brains, *Wnt8b* expression appears to extend upto the pallial-subpallial boundary, suggesting that lateral cortical fates are missing (Fig. 2d). We therefore proceeded with these double conditional mutants for further analysis of the nature of this apparent lateral-to-medial transformation upon loss of both *Lhx2* and *Pax6*. Furthermore, because the boundary between the dorsal and ventral telencephalon is difficult to identify accurately in the double mutant by morphology alone, we used *Dbx1* expression to identify it in subsequent experiments. *Dbx1*, a marker of the antihem [13, 14] is expressed in a subpopulation of scattered cells in the ventricular zone of this structure [15].

## The hem expands to a greater extent upon loss of both *Lhx2* and *Pax6* than upon loss of *Lhx2* alone

**Fig. 3:**
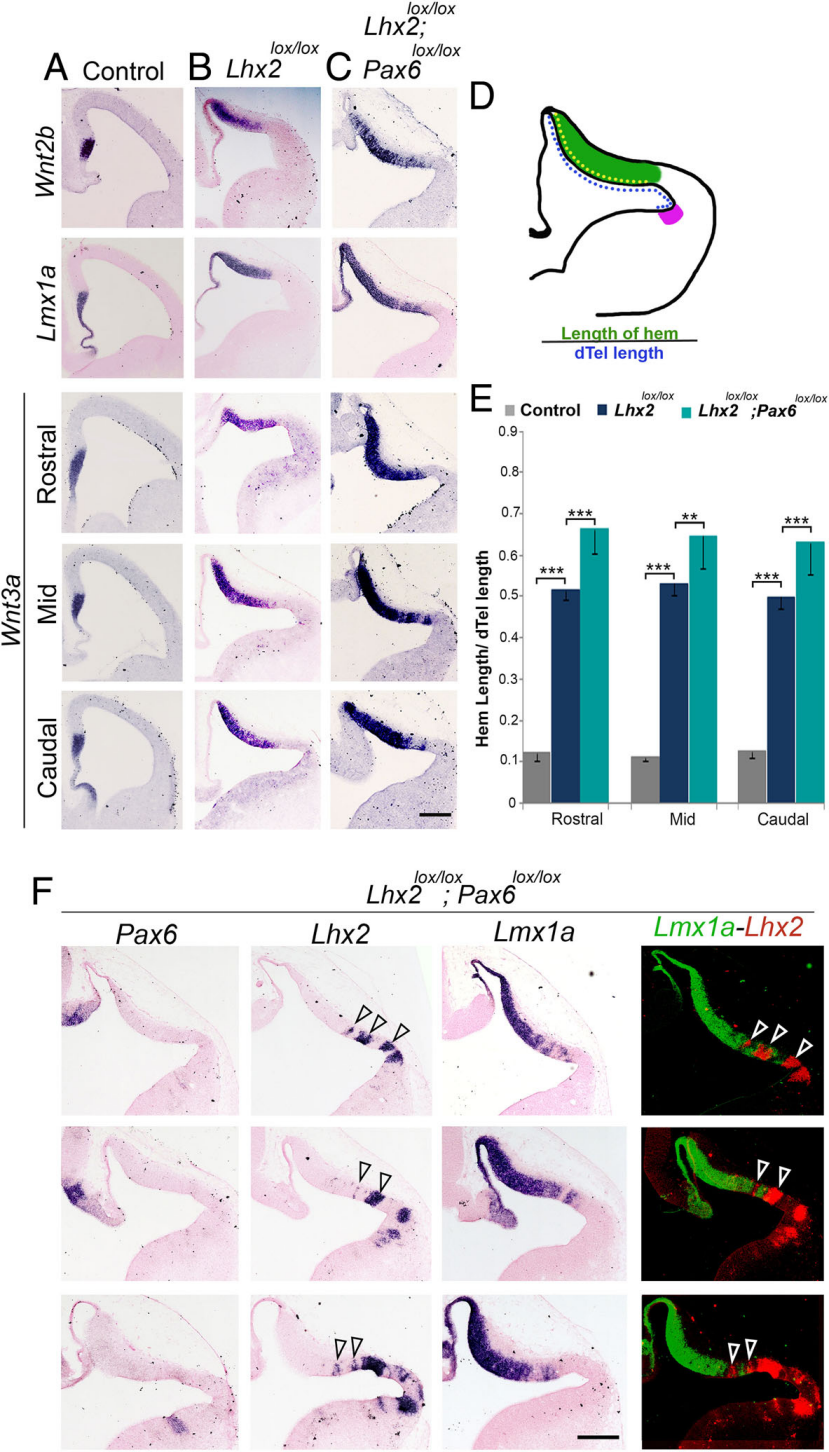
PAX6 restricts the lateral extent to which the hem can expand when LHX2 is lost. (**a**-**c**; **f**) Tamoxifen was administered at E8.5 to Control (**a**), *CreER;Lhx2* ^*lox/lox*^(**b**), *and CreER;Lhx2* ^*lox/lox*^ *;Pax6* ^*lox/lox*^ (**c**, **f**) animals, and the embryos were harvested at E12.5. Coronal sections were examined for the expression of hem markers *Wnt2b* and *Lmx1a*. *Wnt3a* was examined at rostral, mid, and caudal levels of sectioning. In each condition, the length of the hem was scored as a fraction of the total length of the dorsal telencephalic neuroepithelium measured up to the ventral extent of *Dbx1* expression in an adjacent section (shown in Additional file 3: Figure S3). **d** cartoon illustrating the length measurement (yellow dotted line) of the hem (green). Blue dotted line indicates the length measurement of the dorsal telencephalic neuroepithelium measured upto the ventral extent of antihem (pink). **e** Quantification of the fractional length of the hem in control *Lhx2* single conditional mutants, and *Lhx2;Pax6* double conditional mutants, at rostral, mid, and caudal levels of sectioning. For each level, the single mutant hem was significantly longer than that in the control, and the double mutant hem was significantly longer than that in the single mutant or the control (**p < 0.005, ***p < 0.0005). **f** Adjacent sections from three different double conditional mutant embryos in which *Lhx2* was incompletely recombined were examined. In each case, when *Pax6* is completely removed, but *Lhx2* is present in patches (open arrowheads), the hem is suppressed in *Lhx2* expressing regions. A false-color overlay shows that hem (green) is not seen in patches that show *Lhx2* expression (red; open arrowheads). Scale bar is 200 μm

Since loss of *Pax6* alone did not expand medial fates (Fig. 2), we focused only on *Lhx2* single and *Lhx2;Pax6* double conditional mutants for further analysis. We administered tamoxifen at E8.5 to *CreER; Lhx2* ^*lox/lox*^ single conditional mutant mice and to *CreER; Lhx2* ^*lox/lox*^ *;Pax6* ^*lox/lox*^ double conditional mutant mice and harvested the embryos at E12.5. We used a set of three markers, *Wnt2b, Wnt3a*, and *Lmx1a*, to examine the extent of the hem in these mice. All three markers reveal a greater expansion of the hem upon loss of both *Lhx2* and *Pax6* compared with that seen upon loss of *Lhx2* alone (Fig. 3). We quantified the extent of hem expansion by examining *Wnt3a* expression in control, *Lhx2* single conditional mutants, and *Lhx2;Pax6* double conditional mutants (Fig. 3a-d). We calculated the length of the hem as a fraction of the total dorsal telencephalic neuroepithelium measured up to the ventral extent of *Dbx1* expression in an adjacent section (*Dbx1* sections are shown in Additional file 3: Figure S3). We scored rostral, medial, and caudal levels separately and found that there was no significant rostro-caudal difference in the fractional hem length in a given mutant. As is apparent from the images, the hem is longer in *Lhx2* single conditional mutants than in control brains. The key comparison of the *Lhx2* single with the *Lhx2;Pax6* double conditional mutants reveals that the fractional length of the hem is significantly greater when both factors are lost compared with the loss of *Lhx2* alone (Fig. 3e). This indicates that PAX6 functions to restrict the lateral expansion of the hem in an *Lhx2* mutant background.

## LHX2 suppresses hem fate at the lateral extreme of the dorsal telencephalon in a *Pax6* mutant background

Tamoxifen-driven Cre recombination is sometimes incomplete and results in patches of tissue that escape recombination. When we examined the lateral telencephalon of embryos that displayed complete recombination of *Pax6* but had some unrecombined patches of *Lhx2*, it was apparent that hem only formed where both genes were lacking (Fig. 3f). That is, the presence of *Lhx2* in a *Pax6* mutant background was enough to suppress hem fate. This demonstrates that LHX2 is a suppressor of hem fate both medially and laterally, the latter function being revealed only in a *Pax6* mutant background.

In summary, these results demonstrate that the extreme medial fate of the cortical hem can be generated at extreme lateral positions, and that LHX2 and PAX6 each suppresses hem fate. These roles of LHX2 and PAX6 are revealed only upon analysis of the double mutants, and could not have been predicted from examining either single mutant alone.

## Absence of *Lhx2 and Pax6* partially restores the pallial-subpallial boundary/antihem that is disrupted upon loss of *Pax6* alone

**Fig. 4:**
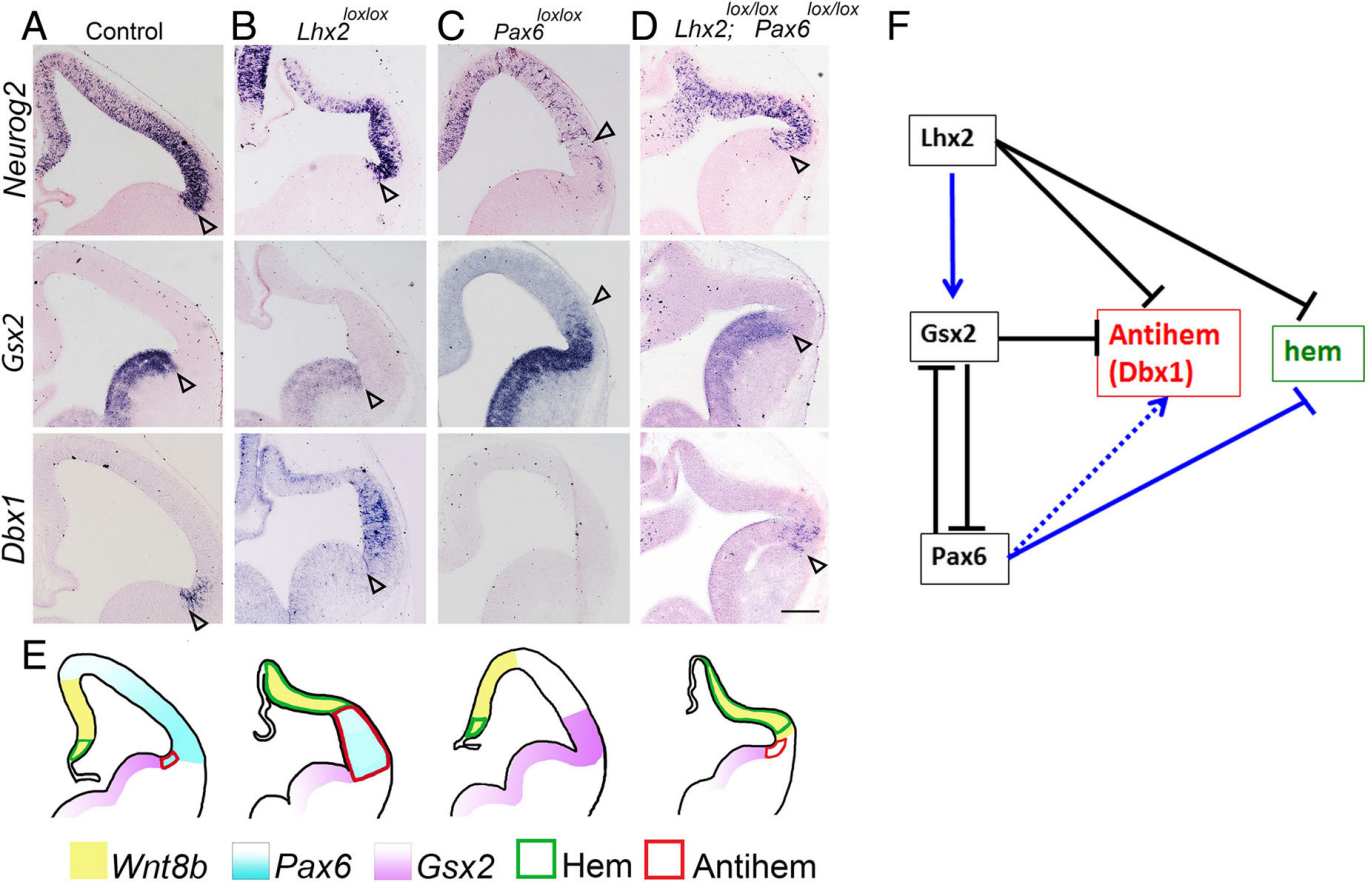
The dorsal shift of the pallial-subpallial boundary upon loss of PAX6 is restored when LHX2 is also removed. **a**-**d** Tamoxifen was administered at E8.5 to Control (**a**), *CreER;Lhx2* ^*lox/lox*^ (**b**), *CreER;Pax6* ^*lox/lox*^ (**c**), *and CreER;Lhx2* ^*lox/lox*^ *;Pax6* ^*lox/lox*^(**d**) animals, and the embryos were harvested at E12.5. Coronal sections were examined for the expression of pallial marker *Neurog2*, subpallial markers *Gsx2,* and antihem marker *Dbx1*. The pallial-subpallial boundary (open arrowheads, **a**-**d**) appears to be normally positioned upon loss of LHX2 alone (**b**), but shifts dorsally upon loss of PAX6 alone, and *Dbx1* expression is lost (**c**). Upon combined loss of both LHX2 and PAX6, the pallial-subpallial boundary appears to be restored to its normal position, together with *Dbx1* expression. Loss of LHX2 results in lowered expression of *Gsx2* in the subpallium compared with control brains (**a**, **b**, **d**). **e** Cartoons schematizing the results in **a**-**d**. **f** A model of the interactions between *Lhx2*, *Gsx2*, and *Pax6* upon the hem and the antihem. Solid blue lines indicate regulatory functions identified in this study: LHX2 maintains *Gsx2* expression levels in the ventral telencephalon, and PAX6 suppresses hem fate in the dorsolateral telencephalon. The dotted blue line indicates that regulation of *Dbx1* by PAX6 is likely to be indirect. Scale bar is 200 μm

Finally, we examined the pallial-subpallial boundary itself, since PAX6 is known to be a regulator of the position of this border [12, 16]. We administered tamoxifen at E8.5 to conditionally remove *Lhx2* alone, *Pax6* alone, or both, and harvested the embryos at E12.5. The expression of both *Neurog2* and *Gsx2* stops at the pallial-subpallial boundary, with *Neurog2* being expressed in the pallial side and *Gsx2* in the subpallial side (Fig. 4, [8]). Loss of *Lhx2* does not appear to alter these expression boundaries, though it does appear to cause an overall reduction in the level of *Gsx2* expression in the subpallium (Fig. 4). In the absence of *Pax6*, the pallial-subpallial boundary is known to shift dorsally, with *Gsx2* expression seen in the lateral region of the pallium where *Neurog2* expression recedes (Fig. 4; [8, 16]). This is thought to be due to cross-repression between PAX6 and GSX2 [8, 13]. However, since LHX2 appears to be required for normal *Gsx2* expression, when *Lhx2* and *Pax6* are both lost, the expanded *Gsx2* expression in the lateral neuroepithelium is no longer seen, but instead, *Neurog2* is expressed there. Thus, despite the absence of *Pax6*, the additional loss of *Lhx2* appears to restore the pallial-subpallial boundary.

This regulation of the pallial-subpallial boundary has an intriguing effect on *Dbx1* expression. GSX2 is known to suppress *Dbx1* and prevent it from being expressed in the subpallium, and consistent with this, *Dbx1* expands into the subpallium in *Gsx2* mutants [15]. Upon loss of *Pax6*, *Gsx2* expression occupies the region of the antihem and suppresses *Dbx1* expression there [15]. Thus, the loss of *Dbx1* expression in the antihem of *Pax6* mutants is likely to be indirect, via GSX2. LHX2 also suppresses *Dbx1* expression [4] and promotes *Gsx2* expression (the current study). The expansion of *Gsx2* into pallial territory upon loss of *Pax6* is reversed upon the additional loss of *Lhx2*. The reappearance of *Dbx1* in its apparently normal antihem location in the *Lhx2* ^*lox/lox*^ *;Pax6* ^*lox/lox*^ double conditional mutant brain may be because both its suppressors- LHX2 or GSX2- are absent in this location.

Therefore, though *Dbx1* expression is undetectable in the *Pax6* single mutant, it is clear from its reappearance (albeit in an ectopic location) in the *Pax6* ^*sey/sey*^ *;Gsx2* ^*−/−*^ double null mutant [15], and in an apparently normal location in the *Lhx2* ^*lox/lox*^ *;Pax6* ^*lox/lox*^ double conditional mutant (this study), that *Dbx1* does not require PAX6, per se*,* for its expression. Like *Dbx1, sFrp2* expression is also lost in the *Pax6* ^*sey/sey*^ brain [15]. However, in contrast to *Dbx1*, we find that *sFrp2* does not reappear in the *Lhx2* ^*lox/lox*^ *;Pax6* ^*lox/lox*^ double mutant (Additional file 4: Figure S4). Since loss of *Lhx2* alone does not cause loss of *sFrp2* expression [17], this suggests that *sFrp2* critically requires PAX6 for its expression. This indicates that different molecular features of the antihem are regulated by distinct combinations of factors, underscoring the heterogeneity of this structure.

## Conclusion

This study uncovers new roles for transcription factors LHX2 and PAX6 in patterning neuroepithelial domains in the telencephalon. LHX2 was known to suppress hem fate in the medial component of the dorsal telencephalon, but we find that PAX6 and LHX2 together suppress hem fate in the lateral component of the dorsal telencephalon as well. We discover that hem fate can indeed arise at the lateral extreme of the dorsal telencephalon, almost upto the pallial-subpallial boundary, if both PAX6 and LHX2 are removed. Therefore, this demonstrates that PAX6 plays a role in suppressing hem fate. Finally, we show that the LHX2 plays a role in regulating the pallial-subpallial boundary. This is surprising since LHX2 is expressed in a gradient in the entire telencephalic ventricular zone, and does not display an expression boundary in the lateral telencephalon. This role for LHX2 is partly due to its regulation of *Gsx2* in the ventral telencephalon, which is a novel genetic interaction between these two developmental control molecules.

Our findings are summarized in the model in Fig. 4, which illustrates how LHX2 and PAX6 interact to regulate the positioning of the hem, the expression of the antihem marker *Dbx1* and also where the pallial-subpallial boundary is formed. These roles for LHX2 and PAX6 are revealed only upon analysis of the double mutants, and could not have been predicted from examining either single mutant alone, thus highlighting the importance of these “classical” epistasis studies in the developing brain. More broadly, an approach that examines the functions of specific factors in the context of different mutant backgrounds may reveal unexpected spatiotemporal interactions between genes and could raise new hypotheses for the control of developmental phenomena. This may be particularly useful in understanding disorders such as autism or other neurodevelopmental diseases in which the severity of the phenotype might depend on the combinations and levels of proteins that may not appear likely to interact with each other based on standard single gene mutation studies.

## Material and methods

### Mice

All animal protocols were approved by the Institutional Animal Ethics Committee (Tata Institute of Fundamental Research, Mumbai, India) according to regulations devised by the Committee for the Purpose of Control and Supervision of Experiments on Animals (CPCSEA), India. The tamoxifen-inducible *CreERT2* line (strain name: B6; 129-Gt(ROSA)26Sortm1(*Cre/ERT*)Nat/J; stock number: 004847) was obtained from the Jackson Laboratory. The *Lhx2* ^*−/−*^ line used in this study was a kind gift from Forbes D. Porter, NIH, USA [9]. The *Lhx2* ^*lox/lox*^ line used in this study was a kind gift from Edwin Monuki, University of California, Irvine, USA [4]. The *Pax6* ^*lox/lox*^ line was a kind gift from David Price, University of Edinburgh [18] and the *Pax6* ^*sey/sey*^ line was a kind gift from Anastassia Stoykova, MPI Goettingen, Germany [12].

Noon of the day of vaginal plug was designated as embryonic day 0.5 (E0.5). Tamoxifen (Sigma, MO, USA) was administered to the pregnant dams at different time points as mentioned in the text and embryos were harvested at E12.5. Control embryos were littermates with one wild-type copy of the relevant gene. The tamoxifen dosage administered was 75 μg/g body weight.

### Sample preparation and in situ hybridization

Freshly harvested brains were fixed in 4% paraformaldehyde (PFA) (Sigma, MO, USA) overnight and equilibrated in 15% sucrose followed by 30% sucrose (SRL Chem, Mumbai, India). The brains were sectioned at 16 μm sections using a freezing microtome and mounted on Superfrost Plus slides (Electron Microscopy Sciences, Hatfield, PA, USA). Sections were post-fixed in 4% PFA, washed in Phosphate buffered Saline (PBS), and treated with proteinase K (Sigma, MO, USA) (1μg/ml) at 37° C for 10 mins. One more round of post-fixing and PBS washes was performed, and, the sections were incubated in hybridization buffer (5X SSC, 50% formamide, 1% SDS) containing different antisense RNA probes at 70° C overnight. Probes were prepared using a kit from Roche per the manufacturer’s instructions (Roche, Mannheim, Germany). The next day, after washes with solution X (2X Saline sodium citrate SSC, 50%formamide, 1%SDS) at 70° C, followed by stringent washes with 2X SSC and 0.2X SSC at room temperature, the sections were washed with TBST (10 mM Tris-HCl, pH 7.5, and 150 mM NaCl, 0.1% KCl, 0.5% Tween-20). The slides were then incubated with alkaline phosphatase conjugated anti-Digoxigenin Fab fragments (1:5000, Roche, IN, USA) for 16 h at 4° C. The slides were then washed four times with TBST and then with developing buffer NTMT (100 mM NaCl, 100 mM Tris, pH 9.5, 50 mM MgCl, 1% Tween-20). The color reaction was performed using Nitro-blue tetrazolium chloride and 5-bromo-4-chloro-3-indolyl-phosphate (NBT-BCIP, Roche, IN, USA) as per manufacturer’s instructions. The incubation was performed for 10–40 h, and terminated when the color reaction had developed satisfactorily, as assessed by the intensity of signal and low background. The reaction was stopped in Tris-EDTA (10 mM Tris-HCl (pH 7.5), 10 mM EDTA (pH 8.0) and fixed 3.7% formaldehyde (diluted in PBS from a 37% stock, Sigma for 1 h at RT. Finally, the slides were washed in PBS, dried and mounted in DPX mountant (S.D Fine Chem, Mumbai, India).

To identify unrecombined cells, an RNA probe against *Lhx2 exon2/3* and *Pax6 exon 5/5a/6* were made by PCR followed by in vitro transcription (Roche, Mannheim, Germany) as per the manufacturer’s instructions. The primers used were as follows;

*Lhx2* *exon 2/3 forward: 5′ CGCGGATCCACCATGCCGTCCATCAGC 3′;*

*Lhx2 exon 2/3 reverse: 5′ GGCGTTGTAAGCTGCCAG 3′;*

*Pax6 exon5/5a/6 forward: 5′ TCACAGCGGAGTGAATCAGCTT 3′;*

*Pax6 exon 5/5a/6 reverse: 5′ CACTGGGTATGTTATCGTTG 3′.*

For each embryo, the extent of recombination was examined by in situ hybridization for the recombined exon in one series of sections. All mutant combinations were examined in at least 4 embryos of the relevant genotype.

### Image acquisition and analysis

Bright field images were acquired by Zeiss microscope Axioskop 2plus using a Nikon DS-fi2 camera. Length measurements were obtained using ImageJ software from eight hemispheres (*n* = 8, from 4 brains) of each genotype: control, single *Lhx2* conditional mutants and *Lhx2;Pax6* double conditional mutants. The hem length was measured as the length of neuroepithelium expressing *Wnt3a*, and dorsal telencephalic neuroepithelium length was measured from the medial edge of the hem up to the ventral extent of *Dbx1* expression in an adjacent section (shown in Additional file 3: Figure S3). For each hemisphere, the ratio of the hem length to the total dorsal telencephalic neuroepithelium length was determined at rostral, medial, and caudal levels of sectioning. The mean and standard deviation (SD) were calculated, and one-way ANOVA followed by the Tukey’s post-hoc test was used to determine significance. Statistical analyses were performed using Graph Pad Prism 5. Error bars in the figure indicate standard deviation (SD).

## Additional file

Additional file 1: Figure S1. Constitutive loss of both *Lhx2* and *Pax6* results in severe cranio-facial defects. (A,B) lateral views and (C,D) dorsal views of E12.5 heads from control (A,C) and *Lhx2* ^*−/−*^ *;Pax6* ^*sey/sey*^ double mutant (B,D) embryos. A control embryo shows the presence of optic cups (black arrowhead) and nostrils (white arrowhead), while the double mutant lacks eyes (black asterisk) and displays two protruding structures in place of the nasal mass (open arrowheads), suggestive of an open or cleft palate (JPEG 965 kb)
Additional file 2: Figure S2. Tamoxifen was administered at E8.5 to *CreER;Lhx2* ^*lox/lox*^ *;Pax6* ^*lox/lox*^ animals and the embryos were harvested at E12.5. Near-complete recombination of *Pax6* and *Lhx2* is seen in a rostro-caudal series of sections adjacent to those examined for *Wnt8b* expression. (JPEG 807 kb)
Additional file 3: Figure S3. This figure shows *Dbx1* expression in adjacent sections to the images shown in Fig. 3. These images were used for measurements of the hem and the dorsal telencephalic neuroepithelium, taken upto the ventral extent of *Dbx1* in adjacent sections in *CreERT2; Lhx2* ^*lox/lox*^ (dashed lines, B,D,F) *and CreER;Lhx2* ^*lox/lox*^ *;Pax6* ^*lox/lox*^ (H,J,L) embryos. *Dbx1* expression in the control brain is not shown. Scale bar is 200 μm. (JPEG 856 kb)
Additional file 4: Figure S4. Tamoxifen was administered at E8.5 to *CreER;Lhx2* ^*lox/lox*^ *;Pax6* ^*lox/lox*^ animals and the embryos were harvested at E12.5. *sFrp2* expression is not detectable in the *Dbx1* expressing antihem of the double mutant embryos. (JPEG 913 kb)

## Abbreviations

LIM-HD: LIM homeodomain

## References

[1] Grove EA (1998). The hem of the embryonic cerebral cortex is defined by the expression of multiple Wnt genes and is compromised in Gli3-deficient mice. Development.

[2] Furuta Y, Piston DW, Hogan BL (1997). Bone morphogenetic proteins (BMPs) as regulators of dorsal forebrain development. Development.

[3] Assimacopoulos S, Grove EA, Ragsdale CW (2003). Identification of a Pax6-dependent epidermal growth factor family signaling source at the lateral edge of the embryonic cerebral cortex. J Neurosci.

[4] Mangale VS (2008). Lhx2 selector activity specifies cortical identity and suppresses hippocampal organizer fate. Science.

[5] Bulchand S (2001). LIM-homeodomain gene Lhx2 regulates the formation of the cortical hem. Mech Dev.

[6] Bishop KM, Goudreau G, O'Leary DD (2000). Regulation of area identity in the mammalian neocortex by Emx2 and Pax6. Science.

[7] Bulchand S, Subramanian L, Tole S (2003). Dynamic spatiotemporal expression of LIM genes and cofactors in the embryonic and postnatal cerebral cortex. Dev Dyn.

[8] Toresson H, Potter SS, Campbell K (2000). Genetic control of dorsal-ventral identity in the telencephalon: opposing roles for Pax6 and Gsh2. Development.

[9] Porter FD (1997). Lhx2, a LIM homeobox gene, is required for eye, forebrain, and definitive erythrocyte development. Development.

[10] Hill RE (1991). Mouse small eye results from mutations in a paired-like homeobox-containing gene. Nature.

[11] Grindley JC, Davidson DR, Hill RE (1995). The role of Pax-6 in eye and nasal development. Development.

[12] Stoykova A (1996). Forebrain patterning defects in small eye mutant mice. Development.

[13] Yun K, Potter S, Rubenstein JL (2001). Gsh2 and Pax6 play complementary roles in dorsoventral patterning of the mammalian telencephalon. Development.

[14] Bielle F (2005). Multiple origins of Cajal-Retzius cells at the borders of the developing pallium. Nat Neurosci.

[15] Carney RS (2009). Differential regulation of telencephalic pallial-subpallial boundary patterning by Pax6 and Gsh2. Cereb Cortex.

[16] Stoykova A (2000). Pax6 modulates the dorsoventral patterning of the mammalian telencephalon. J Neurosci.

[17] Vyas A (2003). Paleocortex is specified in mice in which dorsal telencephalic patterning is severely disrupted. J Comp Neurol.

[18] Simpson TI (2009). Normal ventral telencephalic expression of Pax6 is required for normal development of thalamocortical axons in embryonic mice. Neural Dev.

