# Supplementary figures and images for: Novel functions of LHX2 and PAX6 in the developing telencephalon revealed upon combined loss of both genes
Source: Neural Dev. 2017 Nov 15;12:19. doi: 10.1186/s13064-017-0097-y (PMC5688701; doi:10.1186/s13064-017-0097-y)

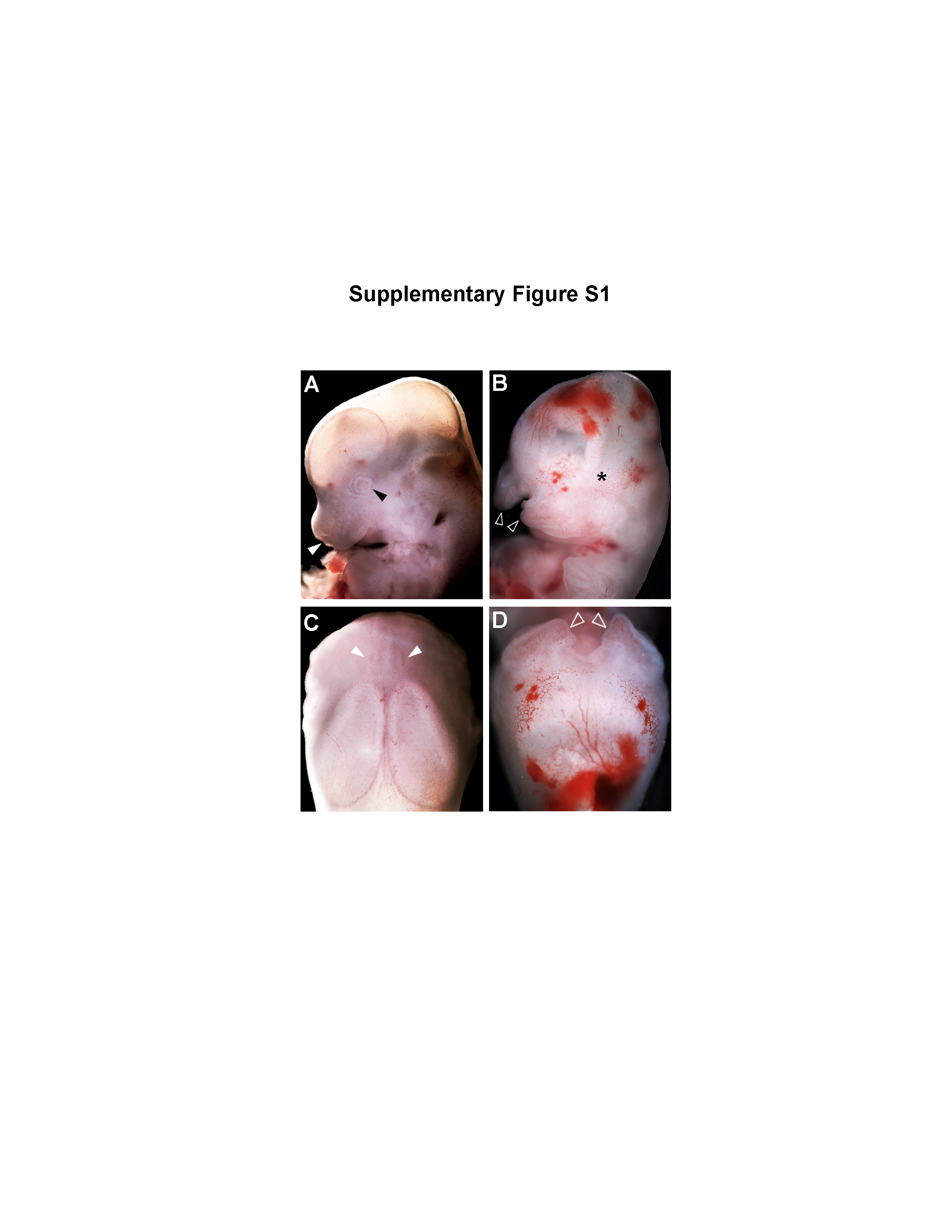

Supplement: Additional file 1: Figure S1. — Constitutive loss of both Lhx2 and Pax6 results in severe cranio-facial defects. (A,B) lateral views and (C,D) dorsal views of E12.5 heads from control (A,C) and Lhx2 −/− ;Pax6 sey/sey double mutant (B,D) embryos. A control embryo shows the presence of optic cups (black arrowhead) and nostrils (white arrowhead), while the double mutant lacks eyes (black asterisk) and displays two protruding structures in place of the nasal mass (open arrowheads), suggestive of an open or cleft palate (JPEG 965 kb) [file 13064_2017_97_MOESM1_ESM.jpg]

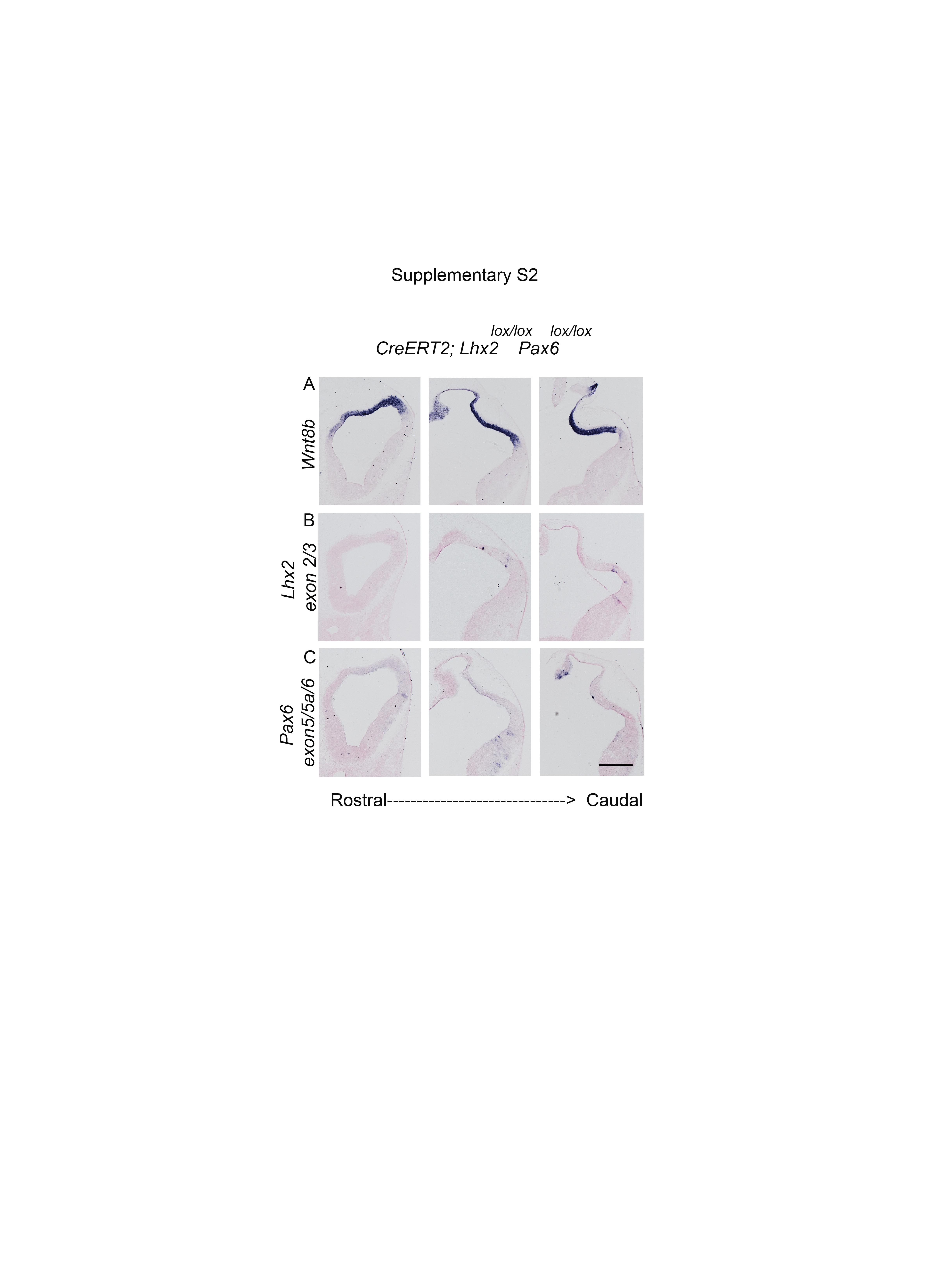

Supplement: Additional file 2: Figure S2. — Tamoxifen was administered at E8.5 to CreER;Lhx2 lox/lox ;Pax6 lox/lox animals and the embryos were harvested at E12.5. Near-complete recombination of Pax6 and Lhx2 is seen in a rostro-caudal series of sections adjacent to those examined for Wnt8b expression. (JPEG 807 kb) [file 13064_2017_97_MOESM2_ESM.jpg]

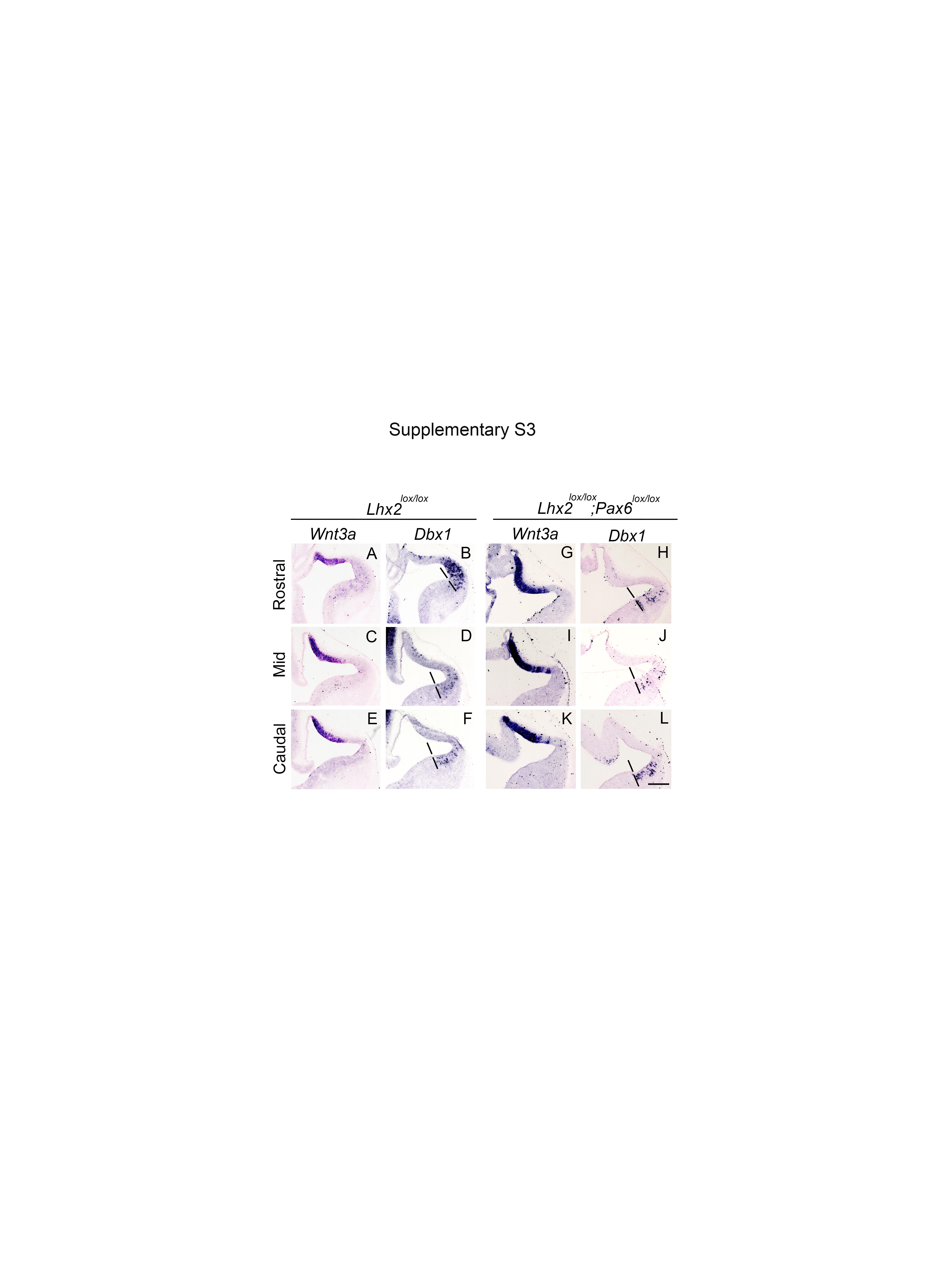

Supplement: Additional file 3: Figure S3. — This figure shows Dbx1 expression in adjacent sections to the images shown in Fig. 3. These images were used for measurements of the hem and the dorsal telencephalic neuroepithelium, taken upto the ventral extent of Dbx1 in adjacent sections in CreERT2; Lhx2 lox/lox (dashed lines, B,D,F) and CreER;Lhx2 lox/lox ;Pax6 lox/lox (H,J,L) embryos. Dbx1 expression in the control brain is not shown. Scale bar is 200 μm. (JPEG 856 kb) [file 13064_2017_97_MOESM3_ESM.jpg]

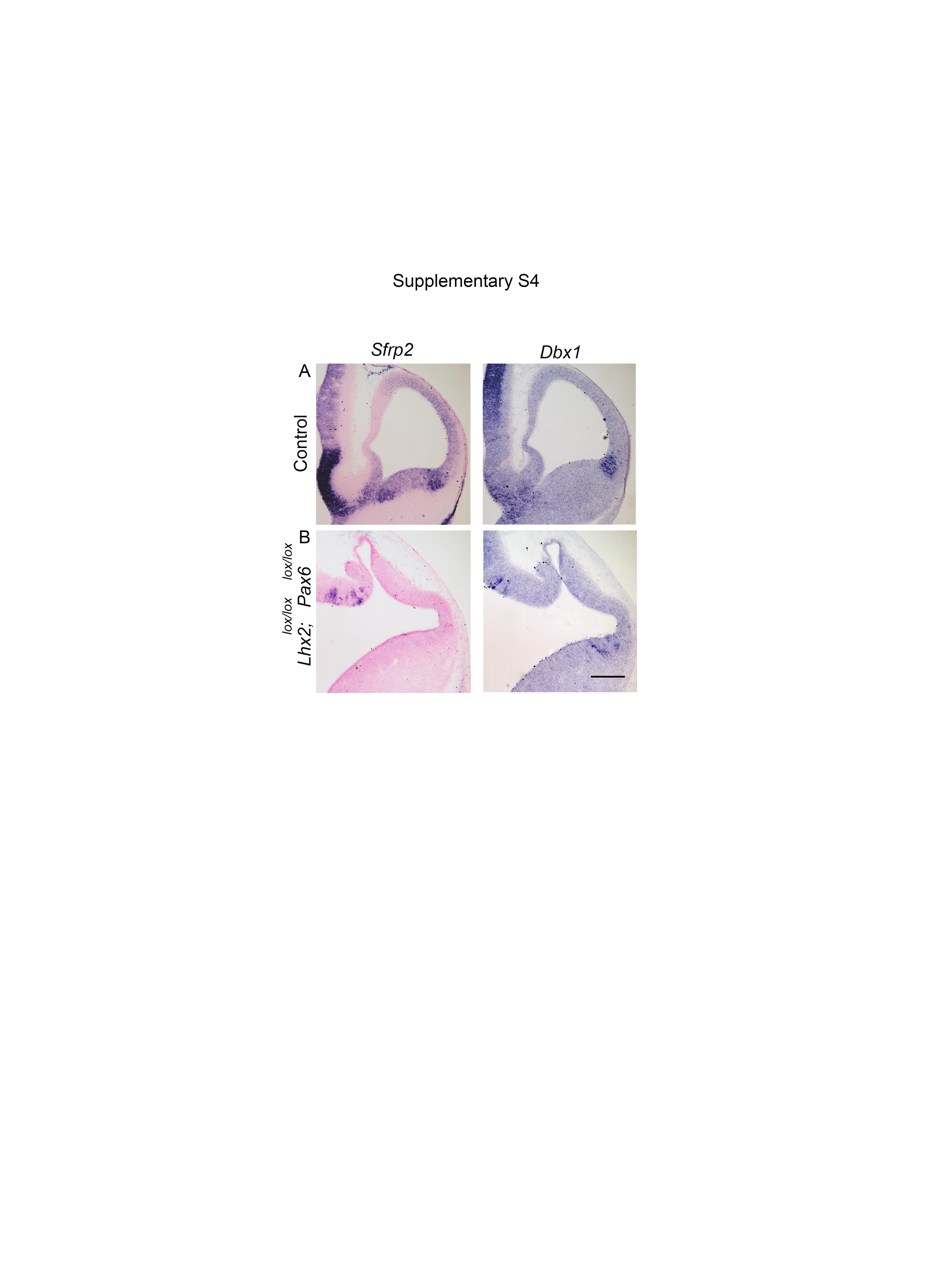

Supplement: Additional file 4: Figure S4. — Tamoxifen was administered at E8.5 to CreER;Lhx2 lox/lox ;Pax6 lox/lox animals and the embryos were harvested at E12.5. sFrp2 expression is not detectable in the Dbx1 expressing antihem of the double mutant embryos. (JPEG 913 kb) [file 13064_2017_97_MOESM4_ESM.jpg]
